# Increased risk for dementia in neurofibromatosis type 1

**DOI:** 10.1038/s41436-021-01261-3

**Published:** 2021-07-13

**Authors:** Roope A. Kallionpää, Mikko Valtanen, Kari Auranen, Elina Uusitalo, Juha O. Rinne, Sirkku Peltonen, Juha Peltonen

**Affiliations:** 1 Institute of Biomedicine, University of Turku, Turku, Finland; 2 Department of Dermatology and Venereology, University of Turku, Turku, Finland; 3 Department of Dermatology, Turku University Hospital, Turku, Finland; 4 Department of Mathematics and Statistics, University of Turku, Turku, Finland; 5 Department of Clinical Medicine, University of Turku, Turku, Finland; 6 Division of Clinical Neurosciences, Turku University Hospital, Turku, Finland; 7 Turku PET Centre, University of Turku, Turku, Finland; 8 Department of Dermatology and Venereology, Institute of Clinical Sciences, Sahlgrenska Academy, University of Gothenburg, Gothenburg, Sweden; 9 Department of Dermatology and Venereology, Region Västra Götaland, Sahlgrenska University Hospital, Gothenburg, Sweden

## Abstract

**Purpose:**

To determine the risk for dementia in neurofibromatosis type 1 (NF1) using a Finnish nationwide cohort of individuals with NF1, and data from national registries.

**Methods:**

A Finnish cohort of 1,349 individuals with confirmed NF1 according to the US National Institutes of Health (NIH) diagnostic criteria was compared with a control cohort of 13,870 individuals matched for age, sex, and area of residence. Dementia-related hospital visits were retrieved from the Finnish Care Register for Health Care using International Classification of Diseases, 10th revision (ICD-10) diagnosis codes G30 and F00–F03. Purchases of antidementia drugs were queried with Anatomical Therapeutic Chemical (ATC) classification code N06D from the drug reimbursement register maintained by the Social Insurance Institution of Finland. The follow-up spanned 1998–2014.

**Results:**

Totals of 16 and 165 individuals with at least two dementia-related diagnoses or drug purchases were identified in the NF1 and control cohorts, respectively. The hazard ratio for dementia in NF1 was 1.67 (95% confidence interval [CI] 1.00–2.80, *P* = 0.050). In an analysis stratified by the type of dementia, the risk for Alzheimer disease was increased in NF1 compared to controls with a hazard ratio of 2.88 (95% CI 1.47–5.66, *P* = 0.002).

**Conclusion:**

Dementia and especially Alzheimer disease are previously unrecognized neurological complications of NF1.

## INTRODUCTION

Neurofibromatosis type 1 (NF1, OMIM 162200) is a dominantly inherited multiorgan syndrome caused by pathogenic variants of the *NF1* gene. The diagnosis of NF1 is often based on symptoms detectable by inspection, most notably cutaneous neurofibromas and café-au-lait macules [1]. The syndrome is also associated with, e.g., highly increased risk for cancer, bone deformities, and hypertension [2]. The prevalence of NF1 is 1/2,000–3,000, and the syndrome causes excess mortality throughout the lifespan [3]. Due to the early mortality associated with NF1, little is known about comorbidities of NF1 among elderly individuals.

Dementia causes major disease burden among the elderly, with the most common type of dementia being Alzheimer disease [4]. Risk factors of dementia include short education, hypertension, and physical inactivity [5]. Drug treatment with anticholinesterases or memantine should be considered already at the diagnosis of dementia, as pharmacotherapy has shown benefit at all disease stages [4].

Cognitive and behavioral problems such as impairment of general cognition and reduced IQ are common in NF1 [6–8]. Individuals with NF1 also have lower educational attainment than matched controls [9, 10]. We have previously reported over threefold standardized mortality ratio for dementia in NF1 [11]. In addition, a recent study observed a nonsignificantly increased risk for dementia-related hospitalizations in NF1 [12]. However, the risk for dementia in NF1 has been poorly recognized [13].

## MATERIALS AND METHODS

The Finnish NF1 cohort was collected by searching all NF1-related hospital visits in the 15 Central and 5 University Hospitals in mainland Finland from 1987 to 2011 [11]. The medical records of the patients were reviewed to confirm NF1 diagnoses according to the NIH diagnostic criteria, resulting in 1,410 individuals with NF1. For each individual with NF1, ten control persons matched for age, sex, and municipality were retrieved from the Finnish Population Register Centre, yielding a control cohort of 14,017 individuals.

The Finnish Institute for Health and Welfare maintains the Care Register for Health Care, which collects information on inpatient care and specialized outpatient care. Dementia-related diagnoses were retrieved using the International Classification of Diseases, 10th revision (ICD-10) diagnosis codes F00 (Dementia in Alzheimer disease), F01 (Vascular dementia), F02 (Dementia in other diseases), F03 (Unspecified dementia), and G30 (Alzheimer disease). Both primary and supplementary diagnoses from inpatient care and specialized outpatient care were considered. The Social Insurance Institution of Finland records purchases of prescription drugs in a register that was queried for Anatomical Therapeutic Chemical (ATC) classification code N06D (antidementia drugs) and the subcodes N06DA (anticholinesterases) and N06DX (other antidementia drugs). History of hypertension was retrieved with ICD-10 codes I10–I15 and ATC codes C02, C08 and C09. History of psychological, behavioral or emotional disorder or mental retardation was searched for using ICD-10 codes F70–F98, and epilepsy was identified with ICD-10 code G40. Information on educational level and causes of death was obtained from Statistics Finland. The dates of death and emigration were from the Finnish Population Register Center. History of cancer was retrieved from the Finnish Cancer Registry.

The follow-up of individuals with NF1 started on the latter of 1 January 1998 or the first NF1-related hospital visit. For control persons, the cohort entry of the respective individual with NF1 was used. The follow-up ended at the second dementia-related encounter, i.e., diagnosis or drug purchase, depending on analysis; death; emigration; or end of study period on 1 January 2015, whichever came first. At least two encounters of interest were required to exclude misregistered data. In an analysis focusing on new diagnoses, the start of follow-up was delayed for one year (washout period) and persons with a dementia-related encounter before the start of follow-up were excluded. Cox proportional hazards models with delayed entry and age as the time scale were used for comparisons of individuals with and without NF1. A frailty term was included to account for matching of controls to individuals with NF1. Dementia-specific survival after dementia diagnosis was assessed using Kaplan–Meier estimates and Cox proportional hazards model. The proportional hazards assumption of the Cox models was assessed using scaled Schoenfeld residuals, and no violations were found. All analyses were carried out using R Software, version 4.0.0.

## RESULTS

Totals of 1,349 individuals with NF1 and 13,870 controls contributed follow-up time during the study period 1998–2014 (Table 1). During the follow-up time, 23 individuals with NF1 and 191 controls had hospital visits related to dementia or Alzheimer disease, or purchases of antidementia drugs. After limiting the analysis to those with at least two dementia-related hospital visits or drug purchases, the case numbers were 16 and 165 among individuals with NF1 and controls, respectively (hazard ratio [HR] 1.67, 95% confidence interval [CI] 1.00–2.80; Table 2). Two individuals with NF1 and seven controls were aged ≤65 years at the first dementia-related hospital visit or drug purchase. The effect of NF1 on the risk for dementia did not differ statistically significantly by age group (≤65 vs. >65) or sex. Thirteen of 16 individuals with NF1 and dementia, Alzheimer disease, or antidementia medication (81%) had history of hypertension, while this was true for 118/165 controls (72%). Among individuals with NF1 > 65 years of age, hypertension was not more common among those with than without dementia (*P* = 0.26). Among those with dementia-related diagnosis or medication, epilepsy was observed in 4/16 individuals with NF1 (25.0%) and 10/165 controls (6.1%). Epilepsy was more frequent among those with than without dementia in both individuals with NF1 and controls (*P* = 0.020 and *P* < 0.001, respectively). The diagnosis of dementia or Alzheimer disease was not preceded by cancer nor a diagnosis of psychological, behavioral or emotional disorder, or mental retardation in any of the individuals with NF1.

**Table 1:** Characteristics of the study cohorts.

	Individuals with NF1	Controls	*P* (NF1 vs. controls)
*n*	1,349	13,870	
Sex			
Females, *n* (%)	694 (51.4)	7,189 (51.8)	0.797
Males, *n* (%)	655 (48.6)	6,681 (48.2)	
Year of birth, mean (SD)	1975.7 (21.6)	1974.9 (22.0)	0.215
Start of follow-up			
Age, mean (SD)	25.7 (20.7)	26.3 (21.0)	0.277
Age 0–19, *n* (%)	661 (49.0)	6,649 (47.9)	
Age 20–39, *n* (%)	320 (23.7)	3,310 (23.9)	
Age 40–64, *n* (%)	315 (23.4)	3,280 (23.6)	
Age ≥ 65, *n* (%)	53 (3.9)	631 (4.5)	
Year, mean (SD)	2001.4 (4.4)	2001.2 (4.3)	0.321
End of follow-up			
Age, mean (SD)	38.3 (20.8)	39.7 (21.5)	0.019
Age 0–19, *n* (%)	320 (23.7)	3,108 (22.4)	
Age 20–39, *n* (%)	449 (33.3)	4,472 (32.2)	
Age 40–64, *n* (%)	403 (29.9)	4,171 (30.1)	
Age ≥ 65, *n* (%)	177 (13.1)	2,119 (15.3)	
Year, mean (SD)	2014.0 (3.0)	2014.6 (1.9)	<0.001
Follow-up time (person-years)			
Sum	17,072.1	185,923.7	
Mean (SD)	12.7 (4.9)	13.4 (4.5)	<0.001
Deaths during the follow-up			
*n* (%)	179 (13.3)	722 (5.2)	<0.001
Age, mean (SD)	56.0 (20.4)	67.3 (17.0)	<0.001
Educational level at the end of follow-up			
ISCED 0–2 (≤9 years of education), *n* (%)	577 (42.8)	5,251 (37.9)	<0.001
ISCED 3–4 (12 years of education), *n* (%)	606 (44.9)	5,218 (37.6)	
ISCED ≥ 5 (≥15 years of education), *n* (%)	166 (12.3)	3,401 (24.5)	

All individuals contributing follow-up time are included in the table irrespective of age.

*ISCED* International Standard Classification of Education, *NF1* neurofibromatosis type 1, *SD* standard deviation.

**Table 2:** Incidence of dementia-related diagnoses and drug purchases among persons with neurofibromatosis type 1 (NF1) and matched controls during 1998–2014.

	Individuals with NF1		Controls		
	Events, *n* (males/females)	Age at first dementia-related encounter, years, mean (SD)	Events, *n* (males/females)	Age at first dementia-related encounter, years, mean (SD)	HR (95% CI), *P*
Alzheimer disease, any type of dementia or antidementia drug (ICD-10 G30, F00–F03, ATC N06D)	16 (8/8)	74.2 (9.0)	165 (63/102)	77.6 (7.1)	1.67 (1.00 to 2.80), 0.050
Alzheimer disease (ICD-10 G30)	10 (6/4)	75.6 (10.0)	61 (19/42)	77.3 (7.6)	2.88 (1.47 to 5.66), 0.002
Any type of dementia (ICD-10 F00–F03)	8 (4/4)	76.9 (6.8)	60 (26/34)	77.3 (8.6)	2.42 (1.15 to 5.09), 0.020
Dementia in Alzheimer disease (ICD-10 F00)	6^a^	75.7 (7.8)	26 (7/19)	77.5 (8.6)	4.04 (1.65 to 9.93), 0.002
Vascular dementia (ICD-10 F01)	<3^a^	-	14 (9/5)	76.6 (7.8)	-
Dementia in other diseases classified elsewhere (ICD-10 F02)	<3^a^	-	5^a^	72.1 (3.0)	-
Unspecified dementia (ICD-10 F03)	3^a^	82.2 (1.7)	13 (3/10)	80.0 (8.9)	5.04 (1.39 to 18.28), 0.014
Antidementia drug (ATC N06D)	15 (7/8)	74.9 (9.6)	129 (48/81)	78.2 (6.1)	2.01 (1.18 to 3.44), 0.011
Anticholinesterase (ATC N06DA)	11 (6/5)	75.7 (10.8)	115 (42/73)	78.1 (5.9)	1.62 (0.87 to 3.01), 0.129
Other antidementia drug (ATC N06DX)	8^a^	77.4 (4.8)	59 (19/40)	79.1 (6.3)	2.50 (1.18 to 5.28), 0.017

All subcodes of the ICD-10 and ATC codes were included. An event is defined as the second encounter of interest (hospital visit, drug purchase or either one, depending on analysis) to avoid any coding errors. The mean ages are shown for the first encounter of interest during the follow-up.

*ATC* Anatomical Therapeutic Chemical classification, *CI* confidence interval, *HR* hazard ratio, *ICD-10* International Standard Classification of Diseases—10th revision, *NF1* neurofibromatosis type 1, *SD* standard deviation.

^a^Sex-stratified and total numbers <3 are not shown to protect patient privacy.

When a washout period of one year was used and only new cases were studied, 10 individuals with NF1 and 74 controls had hospital visits related to dementia or Alzheimer disease, or purchases of antidementia drugs. The HR for first-time dementia-related encounters was 2.28 (95% CI 1.17–4.44) in NF1, compared to controls. The age at the first dementia-related diagnosis or drug purchase was not significantly different in NF1 compared to controls (*P* = 0.20).

When the use of antidementia drugs (ATC N06D) and diagnoses of dementia (ICD-10 F00–F03) were studied separately, NF1 was associated with increased relative risk in both analyses (Table 2). Most observations were related to Alzheimer disease (ICD-10 G30), which was observed in 10 individuals with NF1 (HR 2.88, 95% CI 1.47–5.66; Table 2).

Of the 16 individuals with NF1 and 165 controls with dementia, Alzheimer disease, or antidementia medication, 10 and 65 died during the follow-up, respectively. Among individuals with NF1, 5/10 deaths were due to dementia or Alzheimer disease, and these conditions were mentioned in 7/10 death certificates. The corresponding numbers were 21/65 (32%) and 37/65 (57%) among controls. The survival specific to dementia-related causes of death was 75% among individuals with NF1 and 89% among controls five years after the first observed dementia-related diagnosis or drug purchase. The relative rate of death caused by dementia was 2.42 (95% CI 0.90–6.47) in NF1 compared to controls with dementia or Alzheimer disease.

## DISCUSSION

An increased relative risk for dementia was observed among individuals with NF1 compared to matched controls without NF1. While there have been reports on dementia-related excess mortality [11] and statistically nonsignificantly increased number of dementia-related hospitalizations in NF1 [12], the association of NF1 and dementia has not been previously confirmed. We also observed a nonsignificantly increased risk for dementia-related death after dementia diagnosis in NF1, yet this may be related to the overall high mortality in NF1. As expected, most individuals with dementia or Alzheimer disease were older than 65 years in both NF1 and control groups. The mean age at the first dementia-related diagnosis or drug purchase was slightly although not statistically significantly lower among individuals with NF1 than among controls (Table 2), which is concordant with the idea of NF1-associated predisposition to dementia.

Especially the risk for Alzheimer disease was increased among individuals with NF1. NF1 is well known for its extensive effects on the nervous system. There is a solid body of evidence regarding cognitive impairments in children and adolescents with NF1 [6, 7, 14], and NF1 is also associated with reduced educational level [9, 10]. Moreover, NF1 is associated with hypertension [2]. Given the high prevalence of these risk factors of dementia in NF1, the present observations of excess risks for dementia and Alzheimer disease in NF1 are not completely unexpected. Brain structure has been studied especially among children with NF1, and although differences compared to healthy controls have been observed in, e.g., gray matter and white matter volume, no consistent patterns of association with cognitive phenotype have been found [15, 16]. In a whole-brain comparison of adults with and without NF1, differences were reported in several areas, including hippocampus [17], which shows the earliest structural changes in Alzheimer disease.

The limitations of the present study include hospital-based ascertainment of individuals with NF1, which may bias the cohort toward those with more severe disease manifestations. NF1 is associated with multifaceted comorbidities and the patients may receive more medical attention than controls, leading to higher diagnostic sensitivity. Purchases of antidementia drugs alone may not be completely specific to dementia, yet only 4/16 individuals with NF1 had only drug purchases and no dementia-related hospital visits. Moreover, two of these four patients had been granted a special reimbursement for antidementia drugs, which requires the fulfillment of specific criteria for dementia. As a result of the excess mortality associated with the NF1 syndrome [3, 11], the mean age of the Finnish NF1 cohort is rather low, and only 177 individuals with NF1 contributed follow-up time in the age group >65 years (Table 1). On the other hand, the number of individuals ≤65 years in the cohort ensures sufficient statistical power to detect any associations among working age population, yet we only observed two individuals with dementia and NF1 among those ≤65 years of age. Finally, the number of individuals with NF1 and dementia-related hospital visits or drug purchases is rather low in our material, which hampers our ability to dissect the underlying factors of the increased risk for dementia associated with NF1.

The association of dementia and NF1 showcases a novel neural system complication of NF1 and highlights the need for further studies to understand the pathogenesis of dementia in NF1. Future studies need to address the association of cognitive phenotype and NF1-related changes in brain structure in adults with NF1. Establishing the natural history of changes in cognition in individuals with NF1 is another avenue requiring further investigation. The increased risk for dementia in NF1 may warrant consideration of neuropsychological testing or brain magnetic resonance imaging in elderly individuals with NF1 and signs of cognitive deterioration. Brain amyloid imaging with positron emission tomography or determination of cerebrospinal fluid biomarkers could be used to verify the possible Alzheimer process. Early detection of cognitive decline and changes in imaging or cerebrospinal fluid biomarkers allows timely initiation of therapy.

## Data Availability

Data are available upon request for researchers though data access is restricted. Please contact the Finnish National Institute for Health and Welfare, The Social Insurance Institution of Finland and Statistics Finland for permission. Data can be requested from the corresponding author Juha Peltonen: Institute of Biomedicine, University of Turku, Kiinamyllynkatu 10, FI-20520 Turku, Finland; juhpel@utu.fi.
